# Enabling robust and hour-level organic long persistent luminescence from carbon dots by covalent fixation

**DOI:** 10.1038/s41377-022-00767-y

**Published:** 2022-03-29

**Authors:** Kai Jiang, Yuci Wang, Cunjian Lin, Licheng Zheng, Jiaren Du, Yixi Zhuang, Rongjun Xie, Zhongjun Li, Hengwei Lin

**Affiliations:** 1 International Joint Research Center for Photo-responsive Molecules and Materials, School of Chemical and Material Engineering, Jiangnan University, 214122 Wuxi, China; 2 College of Chemistry, Zhengzhou University, 450001 Zhengzhou, China; 3 State Key Laboratory of Physical Chemistry of Solid Surface, Fujian Provincial Key Laboratory of Materials Genome, and College of Materials, Xiamen University, 361005 Xiamen, China

**Keywords:** Optical materials and structures, Applied optics

## Abstract

The first carbon dot (CD)-based organic long persistent luminescence (OLPL) system exhibiting more than 1 h of duration was developed. In contrast to the established OLPL systems, herein, the reported CDs-based system (named m-CDs@CA) can be facilely and effectively fabricated using a household microwave oven, and more impressively, its LPL can be observed under ambient conditions and even in aqueous media. XRD and TEM characterizations, afterglow decay, time-resolved spectroscopy, and ESR analysis were performed, showing the successful composition of CDs and CA, the formation of exciplexes and long-lived charged-separated states. Further studies suggest that the production of covalent bonds between CA and CDs plays pivotal roles in activating LPL and preventing its quenching from oxygen and water. To the best of our knowledge, this is a very rare example of an OLPL system that exhibits hour-level afterglow under ambient conditions. Finally, applications of m-CDs@CA in glow-in-the-dark paints for emergency signs and multicolored luminous pearls were preliminarily demonstrated. This work may provide new insights for the development of rare-earth-free and robust OLPL materials.

## Introduction

Long persistent luminescence (LPL) materials are widely used in anti-counterfeiting measures, alert signs, optoelectronic devices, and medical diagnostics^1–7^. However, LPL materials are currently limited mainly to the traditional inorganic systems of aluminate-, silicate-, and sulfate-based matrices doped with rare-earth elements, such as europium (Eu) and dysprosium (Dy)^4,5^. Although some nonnoble metal ions (e.g., Na^+^, Ca^2+^, Zn^2+^, Ge^4+^, and Mn^2+^) have been used as co-dopants to improve LPL performance^6,7^, these systems also suffer from potential toxicity, poor processability, high cost, and harsh preparation conditions (usually higher than 1000 °C)^1–3^. Moreover, the large particle size, insolubility, and light scattering abilities of inorganic matrices hinder their applications, particularly in the biomedical field^1–7^. To solve these problems, organic afterglow materials have been developed in recent years^8–10^. In contrast to traditional systems, organic afterglow materials are free of toxic elements, color tunable, and easy to process. The afterglow emissions from most organic materials, however, include phosphorescence and thermally activated delayed florescence (TADF) due to intersystem crossing (ISC) or reverse ISC (RISC) between the excited singlet and triplet states, which exhibit relatively long lifetimes with exponential decay^11–13^. Nevertheless, their afterglow durations are usually limited to seconds^8,14,15^ and are not comparable to the hour-level afterglow of inorganic LPL materials.

In contrast to triplet-state-based phosphorescence and TADF, organic LPL (OLPL) generally contains long-lived intermediate states and undergoes power-law decay^1–5^. To achieve OLPL, the production of long-lived intermediates, such as charged-separated (CS) states, is a possible route, and it has been confirmed in certain electron-donating and electron-accepting organic blends^16–18^. In these systems, charge-transfer (CT) states are first formed; photoexcited electrons from the donor molecule are transferred from its lowest unoccupied molecular orbital (LUMO) to the LUMO of the acceptor molecule. Then, the long-lived CS states can form by diffusion and isolation of the donor radical cations into the acceptor radical anions^19–21^. Unfortunately, these reported OLPL systems must be fabricated under a nitrogen atmosphere to prevent quenching of the CT states and CS states from oxygen and water. Therefore, realizing OLPL under ambient conditions remains a great challenge, particularly in aqueous media.

**Fig. 1:**
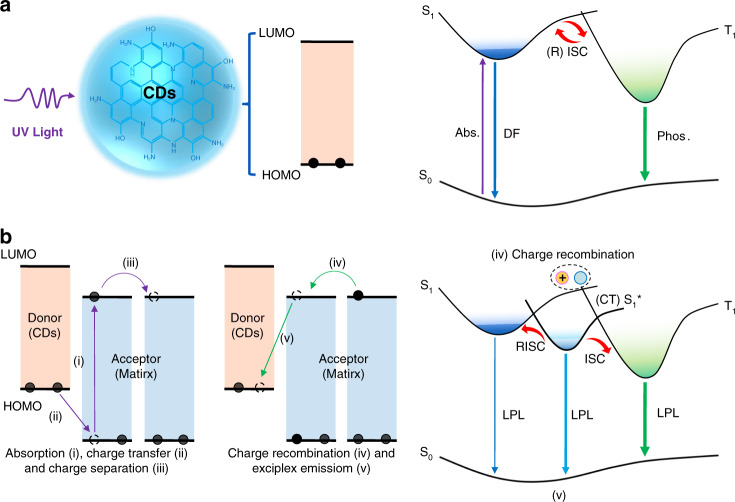
Possible mechanisms for achieving long-lived luminescence from CDs. **a** The emission mechanism of delayed fluorescence (DF) and phosphorescence: upon photoexcitation, electrons are excited from the ground state (S_0_) to the excited singlet state (S_1_) and then undergo intersystem crossing (ISC) to form the excited triplet state (T_1_), from which electrons relax to S_0_ by emitting phosphorescence; alternatively, electrons can return to S_1_ by reverse ISC (RISC) and then relax to S_0_ by emitting DF. Phosphorescence was abbreviated as Phos in this figure for clarity. **b** A possible LPL mechanism: during photoexcitation, electrons (black dots) are continuously generated and filled into the LUMO orbits (path i; the black dashed circle representing possible hop positions of electron, the same below); in the blends of CDs and matrix, electrons are transferred from the HOMO of the donor to the HOMO of the acceptor to form charge-transfer (CT) states (path ii); formation of the charge-separated (CS) states through diffusion and isolation of the donor radical cations (CDs) into the acceptor radical anions (matrix) (path iii); gradual recombination of the radical anions and radical cations (path iv) to generate long persistent exciplex emission, electronic transition from the LUMO of the acceptor to the HOMO of the donor (path v). The small energy gaps between the lowest singlet excited state of exciplex (S_1_*) and S_1_/T_1_ of CDs enable RISC and ISC, resulting in multiple LPL emission processes.

Carbon dots (CDs), a new kind of photoluminescent (PL) material, have attracted much interest in recent years due to their excellent photophysical properties, chemical stability, biocompatibility, and facile preparation^22–24^. More impressively, CDs-based afterglow materials with various performances have also been exploited recently; they have been embedded in specific matrices or themselves containing unique structures^25–36^. However, the afterglow in all the relevant reports is attributed to phosphorescence and/or TADF (Fig. 1a); thus, the afterglow durations are limited to seconds as well^25–36^. Notably, the photoinduced electron transfer properties of CDs have been studied previously, and they have demonstrated that CDs can be used as both electron donors and electron acceptors^37–39^. Given these findings, photoinduced CT and CS states might be obtained by properly designing CD-organic molecule blends, consequently producing LPL (Fig. 1b). To realize such a purpose, the following conditions need to be considered: (i) the energy levels of the CDs and organic molecules should be fit for electron transfer (the highest occupied molecular orbital (HOMO) and LUMO energy levels of the donor should be higher than those of the acceptor)^19–21^; (ii) CDs should be employed as electron donors and immobilized in an appropriate electron-acceptor matrix, providing a rigid microenvironment to stabilize the CT and CS states; and (iii) the formation of covalent bonds is preferred between the CDs and matrix molecule to further stabilize the excited states and prevent quenching by oxygen and water^40,41^.

Following the above mentioned prerequisites and after carefully screening a variety of possible systems, we found a blend of m-CDs (prepared from m-phenylenediamine) and cyanuric acid (CA) that exhibited robust LPL properties (applicable under ambient conditions and even in aqueous media). This blend (named m-CDs@CA) can be facilely fabricated by the microwave-assisted heating of a mixture of m-CDs and urea. Surprisingly, the m-CDs@CA showed an afterglow that lasted longer than an hour when irradiated with a conventional hand-held UV lamp (365 nm). Further studies indicated that CA was produced from urea in situ during the microwave heating process and bonded with the m-CDs via a C–N bond, and the LPL originated from the exciplexes of the m-CDs and CA. The embedding of CDs in CA crystals in situ and the formation of C–N covalent bonds between CA and m-CD were confirmed to play critical roles in rigidifying the microenvironment of the CT and CS states of the exciplexes, thus activating the LPL of m-CDs@CA and preventing its quenching from oxygen and water. To the best of our knowledge, this is a very rare example of an OLPL system that shows hour-level afterglow under ambient conditions and is even applicable in aqueous media. Finally, applications of m-CDs@CA in glow-in-the-dark paints for emergency signs and multicolored luminous pearls were preliminarily demonstrated. This work may provide new insights for the development of robust OLPL materials free of rare-earth metals.

## Results

### Synthesis and structural characterization of m-CDs@CA

**Fig. 2:**
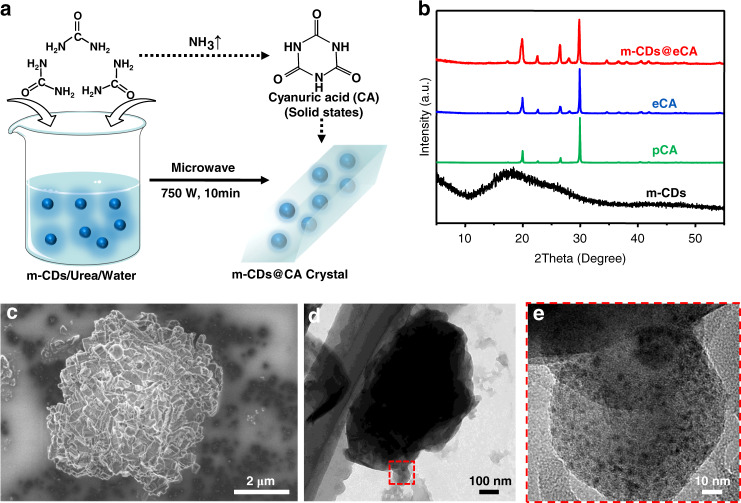
The preparation process and structure characteristics of m-CDs@CA. **a** Schematic illustration of the preparation process of m-CDs@CA and the conversion from urea to CA. **b** XRD patterns of m-CDs, pCA, eCA, and m-CDs@CA. **c–e** High-resolution SEM, TEM, and high-resolution TEM (red area in **d**) images of m-CDs@CA.

The m-CDs-based LPL materials (i.e., m-CDs@CA) were fabricated with various ratios of m-CDs and CA by a facile microwave-assisted heating treatment, in which CA was produced from urea during the heating process and crystallized as the matrix in which the m-CDs were embedded (Fig. 2a, and see supplementary information for details). According to Figs. S1, S2 and Table S1 in the supplementary information, the samples with lower ratios of m-CDs (m-CDs:urea by weight: 0.05% and 0.1%) exhibited better optical performances. To conveniently examine the structural information, the sample of 0.1% m-CDs was discussed in this study. The conversion from urea to CA was confirmed by XRD analysis. As shown in Fig. 2b, the experimental CA (eCA, synthesized from urea by the microwave-assisted method, see details in the experimental section of the supplementary information) and m-CDs@CA displayed XRD patterns similar to those of pure CA (pCA, purchased from J&K Chemical Reagent Co. Ltd, Beijing, China) with only slight differences in some X-ray diffraction peak intensities, demonstrating that fine CA crystals were formed when urea was heated in a microwave in either the presence or absence of m-CDs. The increased intensities of the X-ray diffraction peaks of m-CDs@CA may be due to the effects of stacking and the crystallization orientation of the CA molecules because the m-CDs were embedded in the CA crystals. Moreover, SEM images showed different morphologies of m-CDs@CA and pCA (Fig. 2c and Figs. S3, S4 in the supplementary information), further indicating that embedding the m-CDs in CA affected its crystallization process. The XRD and TEM images show that the m-CDs were spherical, amorphous carbon/polymer structures (black line in Fig. 2b and Fig. S5 in the supplementary information). Significantly, the successful and uniform incorporation of the m-CDs into the CA crystals was clearly confirmed by the TEM and high-resolution TEM images of m-CDs@CA (Fig. 2d and e).

### The long persistent luminescence and charge separation states of m-CDs@CA

**Fig. 3:**
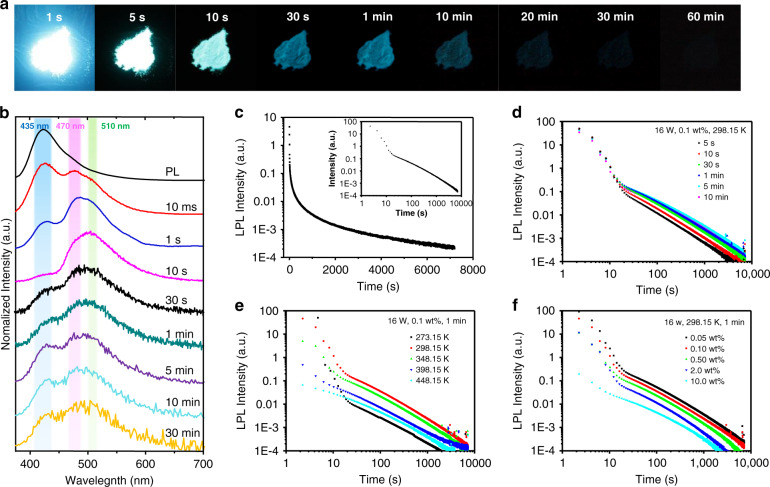
The LPL properties of m-CDs@CA. **a** Photographs of m-CDs@CA powder (0.1 wt% sample) after the irradiation source was removed (excitation wavelength, 365 nm; excitation power, 16 W; excitation time, 1 min; temperature, 298.15 K). **b** The photoluminescence and time-resolved afterglow spectra of the 0.1 wt% m-CDs@CA sample under ambient conditions. The colored areas highlight the range of emission wavelength maxima. **c** The LPL decay profile of the 0.1 wt% m-CDs@CA powder (excitation power, 16 W; excitation time, 1 min; temperature, 298.15 K) and the logarithmic plot of the decay profile (insert). **d–f** Excitation time (**d**), sample temperature (**e**), and ratio of m-CDs to urea (**f**)-dependent LPL logarithmic decay profiles.

The prepared m-CDs@CA displayed bright blue photoluminescence (PL) (emission wavelength maximum (λ_max_) at ~425 nm) with an excitation-independent feature (Fig. S1a in the supplementary information); they were similar to the free m-CDs (Fig. S6 in the supplementary information). This result also indicates that the m-CDs were successfully embedded in the CA crystals and were likely responsible for the observed PL from m-CDs@CA. Surprisingly, the m-CDs@CA had a very long afterglow (longer than one hour) that was observable by the naked eye after the UV irradiation was turned off (Fig. 3a). To further investigate the afterglow of the m-CDs@CA, their steady-state PL and time-resolved afterglow spectra (10 ms, 1 s, 10 s, 30 s, 1 min, 5 min, 10 min, and 30 min after ceasing the irradiation) were obtained, from which apparent spectral transformation (the relative intensity of emission λ_max_ at ~425, 470, and 510 nm changed) was observed (Fig. 3b). Specifically, the emission rapidly decayed at 425 nm, but the relative intensities at 470 and 510 nm increased within 10 s after excitation ceased. The afterglow emission at 425 nm was consistent with the steady-state PL (black line in Fig. 3b) and exhibited typical double-exponential decay behavior (with an average lifetime of 1.26 s, Fig. S7 and Table S2 in the supplementary information), implying the nature of TADF^42,43^. In contrast, from 10 s to 30 min, the time-resolved afterglow spectra also exhibit multiple emission peaks (425, 470, and 510 nm) with relative intensity alterations, demonstrating that multiple long-lived emissive states were produced from the m-CDs@CA under UV irradiation. The afterglow of m-CDs@CA can last more than 2 h if the decay spectrum is measured (Fig. 3c). To the best of our knowledge, this is the first example of hour-scale afterglow from CD-based materials. The afterglow intensity of the m-CDs@CA follows an exponential decay for ~10 s and then obeys the inverse power function of time *t*^*−1*^ (insert of Fig. 3c). An emission decay with an inverse power function of time can be explained using the Debye-Edward law (*t*^*−m*^, with m = 1), in which long-range electron transfer and recombination are described^44^. The Debye-Edward law offers a reasonable explanation for the emission of directly ionized phosphors in frozen solution or immobilized in a host. Such an emission process can be modeled as the diffusion of ejected electrons through the matrix to the radical phosphors^45,46^. In this model, *m* = 1 corresponds to a low concentration of recombination states^21^. Therefore, the *t*^*−1*^ emission decay profile of m-CDs@CA implies the presence of CS intermediate states during UV irradiation, and these states are likely responsible for the observed LPL.

To further confirm the presence of CS-state emission from m-CDs@CA, the excitation time-, sample temperature-, and m-CD content-dependent persistent luminescence were examined in detail. Generally, the duration time of CS-state-based LPL materials is related to the excitation time due to the charge storage mechanism^1–5^. As shown in Fig. 3d, the LPL durations were found to increase with prolonged excitation time; the emission duration lasted only ~2000 s after 5 s of UV excitation, but it lasted more than 7000 s with 1 min of excitation. With a longer excitation time (over 5 min), however, the LPL intensity and duration remained nearly constant; this observation is attributed to the number of accumulated charge carriers that reached the upper limit. The excitation time-dependent LPL duration behavior is additional evidence for the generation and accumulation of charge carriers in m-CDs@CA under photoirradiation^1–3^. In addition, the temperature of the sample would also affect the LPL duration due to the nonradiative deactivation of molecular vibrations^1–3^. As shown in Fig. 3e, the LPL duration increased from 273.15 to 298.15 K and then decreased at temperatures above 298.15 K, suggesting that nonradiative deactivation by molecular vibration could be suppressed at room temperature but increased at higher temperatures. Charge mobility and RISC efficiency are improved at higher temperatures, and both of these factors lead to a decrease in LPL duration as well^21,43^. Then, the contents of m-CDs in m-CDs@CA-dependent LPL durations were investigated. As shown in Fig. 3f, the durations drastically decreased from 7000 s to 2000 s as the ratio of m-CDs increased from 0.05 to 10 wt% (relative to urea). This phenomenon can be explained by the reduced distances between the CS-state species in m-CDs@CA due to the higher contents of m-CDs, leading to an increase in recombination probability^21^. Finally, time-resolved electron spin resonance (ESR) spectra of the m-CDs@CA were measured, and these spectra provided direct evidence for the presence of radicals in the material. As shown in Fig. S8 (in the supplementary information), the ESR signals were observed to obviously increase and then gradually decrease with time after UV light irradiation, indicating the presence of charged species (CS states) in m-CDs@CA.

### The mechanism of long persistent luminescence emission from m-CDs@CA

**Fig. 4:**
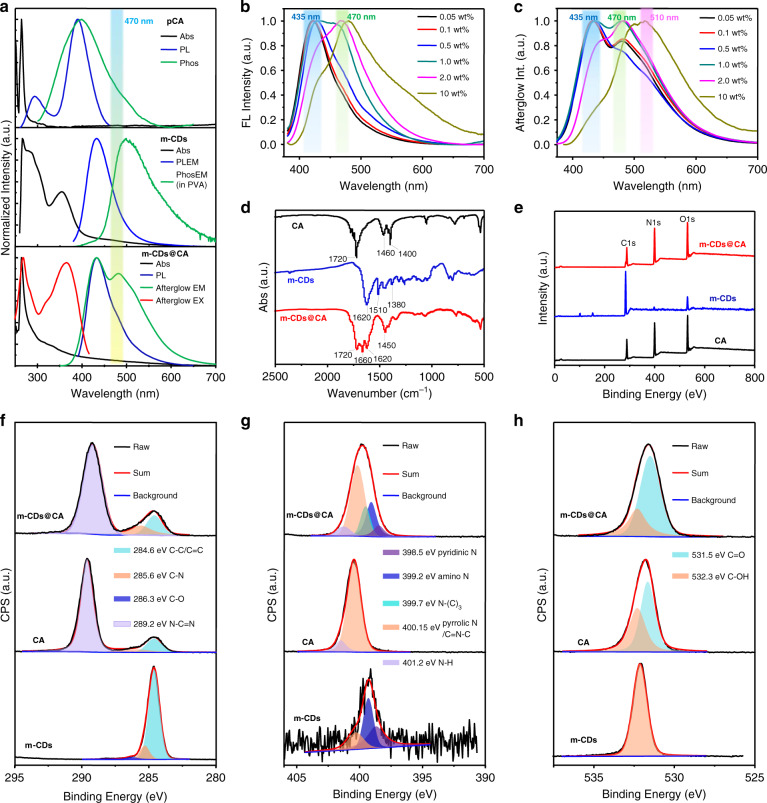
The photophysical properties and chemical composition of CA, m-CDs and m-CDs@CA. **a** UV-Vis absorption (Abs), photoluminescence (PL) emission and excitation spectra of CA (top), m-CDs (middle), and m-CDs@CA (bottom) under ambient conditions. PL, phosphorescence emission and excitation spectra of m-CDs were measured from their ethanol dispersion and PVA film, respectively; PL, afterglow emission and excitation spectra of CA and m-CDs@CA (0.1 wt% sample) are measured from their powders. The absorption spectra of m-CDs, CA and m-CDs@CA were obtained from DMF dispersions of each material. Phosphorescence was abbreviated as Phos in this figure for clarity. **b, c** PL (**b**) and afterglow (**c**) emission spectra of m-CDs@CA with different ratios of m-CDs under 365 nm excitation. **d, e** FT-IR (**d**) and XPS (**e**) spectra of m-CDs, CA, and m-CDs@CA. **f–h** High-resolution XPS spectra and the corresponding fitting curves of the C 1 s (**f**), N 1 s (**g**), and O 1 s (**h**) core levels of the CA, m-CDs, and m-CDs@CA.

To clarify the origins of the LPL emission, UV-Vis absorption, PL and afterglow spectra of m-CDs, pCA, and m-CDs@CA were measured and analyzed. As shown in Fig. 4a, both m-CDs and CA have an absorption peak at ~270 nm, attributed to the π → π* transition of the C=C bond^47,48^. In addition, m-CDs have another absorption band centered at ~350 nm, which can be ascribed to the n→π* transition of C=N relevant moieties in m-CDs^41,49^. PL and afterglow emission λ_max_ of m-CDs (dispersed in PVA film) and CA located at 425 and 506 nm and 380 and 390 nm, respectively (Fig. 4a, top and middle). Unexpectedly, the PL spectrum of m-CDs@CA not only shows a main emission band centered at 425 nm (arising from m-CDs) but also a shoulder emission at ~470 nm (Fig. 4a, bottom). Notably, the relative PL emission intensity of the m-CDs@CA samples at 470 nm gradually increases with increasing m-CD content (Fig. 4b and Fig. S1 in the supplementary information). Consequently, this new PL emission peak at 470 nm could originate from the exciplexes of m-CDs and CA. Similarly, the afterglow emission intensity of the m-CDs@CA samples also gradually increases at 470 nm as the ratios of m-CDs increase from 0.05 to 2.0 wt% (Fig. 4c and Fig. S2 in the supplementary information). At a higher ratio of m-CDs (e.g., 10 wt%), however, the corresponding sample exhibits a predominant afterglow emission located at 510 nm (Fig. 4c and Fig. S2 in the supplementary information), similar to the phosphorescence of the m-CDs. This redshifted afterglow emission might be the result of the ISC of excitons from the excited state of the exciplex to the T_1_ states of the m-CDs, which were then released in the phosphorescence manner. Moreover, the afterglow excitation spectrum of m-CDs@CA, which contains an emission peak at 470 nm, is in good agreement with the main absorption bands at ~270 nm and 360 nm, indicating that the afterglow emission likely originates from both the C=C and C=N structures of the m-CDs. Based on these observations, we can tentatively conclude that excited triplet states and exciplexes are formed from m-CDs@CA under UV light excitation and are likely responsible for their long-lived emissions.

To determine the effects of the embedded form, another m-CD and CA composite (named m-CDs#CA, see details in the experimental section in the supplementary information) was prepared using co-crystallization. In comparison with m-CDs@CA, m-CDs#CA shows very weak exciplex-based emission (i.e., λ_max_ at ~480 nm, Figs. S9 and S10 in the supplementary information), indicating that the formation of an exciplex from this composite is not as efficient as that from m-CDs@CA. Moreover, the afterglow of m-CDs#CA follows typical exponential decays with lifetimes of 1.67 and 1.54 s at 425 and 480 nm, respectively (Fig. S11 and Table S3 in the supplementary information)^41,50^, but no LPL can be observed. The different afterglow properties of m-CDs#CA and m-CDs@CA indicate that the LPL emission from m-CDs@CA is also dependent on the form of the m-CDs embedded in the CA crystals. To clarify this issue, Fourier transform infrared (FT-IR) and X-ray photoelectron spectroscopy (XPS) spectra of m-CDs, CA, and m-CDs@CA were measured and analyzed. s-Triazine-2,4,6-trione is the stable form of CA in the solid state^51,52^, and thus, the characteristic peaks observed at ~1720 cm^−1^ in CA and m-CDs@CA can be attributed to the stretching vibration of the C=O bonds of CA (Fig. 4d). According to a careful comparison of these FT-IR spectra, m-CDs@CA shows increased absorption at 1450 cm^−1^ and emergence of an absorption peak at ~1660 cm^−1^, which likely corresponds to the stretching vibrations of the C–N and C=O bonds of amides, respectively. These findings suggest that chemical reactions occurred during the in situ microwave-assisted formation of m-CDs and CA; this reaction was the production of covalent C–N bonds between the m-CDs and CA. This assumption can be further verified by XPS. The XPS survey spectra showed that the m-CDs@CA, m-CDs, and CA contained the same elements (carbon, nitrogen, and oxygen) (Fig. 4e). The carbon content increased from CA to m-CDs@CA, which further supports the successful incorporation of m-CDs into the CA crystals (Table S4 in the supplementary information). The C 1 s, N 1 s, and O 1 s XPS spectra of the m-CDs@CA, CA, and m-CDs were deconvoluted and fitted (Fig. 4f-h). The C 1 s XPS spectrum of the m-CDs@CA can be fitted with three binding energies at 284.6, 285.5, and 289.2 eV (Fig. 4f), corresponding to the C–C/C=C, C–N, and N–C=N bonds, respectively. The N 1 s XPS spectrum can be deconvoluted into five binding energies at 398.5, 399.2, 399.7, 400.15, and 401.2 eV (Fig. 4g), which are attributed to pyridinic N, amino N, N–(C)_3_, pyrrolic N/C=N–C, and N–H bonds, respectively. The O 1 s XPS spectrum, which showed two components, can be assigned to the C=O (531.5 eV) and C–OH (532.3 eV) bonds (Fig. 4h). To determine the relative alterations of the chemical groups, all the deconvoluted XPS fitting results of m-CDs@CA, m-CDs, and CA were collected and are shown in Table S5 (in the supplementary information). From these results, one can see an increase in C–N but obvious decreases in the N–H and –OH contents from CA to m-CDs@CA based on the C 1 s, N 1 s, and O 1 s XPS fitting results, implying that more C–N bonds formed in the m-CDs@CA and were accompanied by consumption of N–H and –OH functional groups. Moreover, new N-(C)_3_ bonds were present in the m-CDs@CA according to the N 1 s XPS fittings, again confirming the formation of C–N covalent bonds between the m-CDs and CA in m-CDs@CA. In contrast, only hydrogen bonds could form in the co-crystallized sample of m-CDs and CA (i.e., m-CDs#CA)^47^. Therefore, the formation of covalent bonds between m-CDs and CA is critical for activating LPL of m-CDs@CA.

**Fig. 5:**
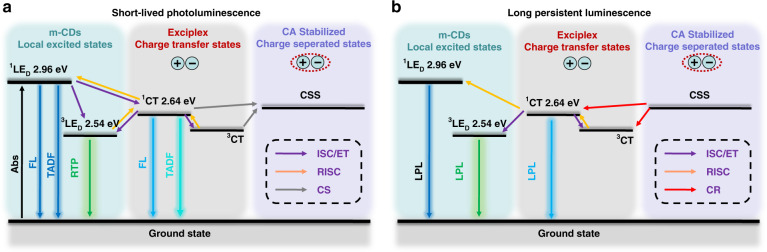
A proposed emission mechanism of m-CDs@CA **a**, **b** The emission mechanism before (i.e., short-lived PL including FL, RTP and TADF) and after recombination of CS states of exciplex (i.e., LPL) of m-CDs@CA under the UV light (365 nm) irradiation. The energy levels were calculated based on the onsets of the corresponding emission spectra. Electron donor (D, i.e., m-CDs), acceptor (A, i.e., CA), absorption (Abs), fluorescence (FL), charge transfer (CT, i.e., exciplex), electron transfer (ET), charge–separated states (CSS), charge separation (CS), charge recombination (CR), thermally activated delayed fluorescence (TADF), room temperature phosphorescence (RTP), and long persistent luminescence (LPL).

To further understand the LPL mechanisms, the low-temperature (77 K) PL and afterglow of m-CDs and m-CDs@CA were investigated (Figs. S12 and S13 in the supplementary information). The photophysical parameters and energy levels were calculated based on the onsets of their emission spectra and summarized in Table S6 (supplementary information). Although the lowest excited triplet state of exciplex (^3^CT) cannot be obtained directly, it is reasonable to assume that the ^3^CT is nearly identical to the lowest excited singlet state of the exciplex (^1^CT) because the exciplex only induces a small energy gap between ^1^CT and ^3^CT due to the good separation of the HOMO and LUMO orbitals on the donor (m-CDs) and acceptor (CA) (Fig. S14 and Table S6 in the supplementary information)^53–55^. The short-lived PL and LPL mechanisms and energy diagram of m-CDs@CA are proposed in Fig. 5; in this mechanism, the m-CDs and CA act as donors and acceptors, respectively, and exciplexes are formed from the m-CDs@CA upon photoexcitation. The ^1^CT of the exciplex is deemed to be responsible for the newly emerged short-lived PL emission band centered at 470 nm; therefore, the energy level of ^1^CT is likely located between the lowest singlet excited state (^1^LE_D_) and the lowest triplet state (^3^LE_D_) of the donor (i.e., m-CDs), corresponding to the emission at 425 and 510 nm, respectively (Fig. 5a). Since strong covalent bonds formed between the m-CDs and CA, they could effectively stabilize the triplet states of the m-CDs. In addition, crystallized CA would also play a role in protecting the excited triplet states of the embedded m-CDs from being quenched by molecular oxygen. Therefore, room temperature phosphorescence (RTP) could occur from m-CDs@CA, but it was not directly observed because it was masked by the LPL signals. Recent studies on TADF materials showed that the locally excited triplet state of the donor or acceptor was similar to the triplet state located on the donor or acceptor molecule in an exciplex^56–58^. The energy gaps between the ^1^CT and ^1^LE_D_ (ΔE(^1^LE_D_-^1^CT)) and the ^1^CT and ^3^LE_D_ (ΔE(^1^CT-^3^LE_D_)) were calculated to be 0.32 and 0.10 eV, respectively, smaller than that between the ^3^LE_D_ and ^1^LE_D_ (ΔE(^1^LE_D_-^3^LE_D_) = 0.42 eV). Thus, ^1^CT might act as an intermediate to activate TADF from emissive ^1^LE_D_ through the RISC process (Fig. 5a). As a result, m-CDs@CA could exhibit TADF and RTP emission, corresponding to the afterglow emission peaks at 425 nm and the shoulder at 510 nm, respectively (Fig. 4a, bottom). However, the TADF lasts only ~10 s, as it decays exponentially. The LPL emission originated from the CT state transition of the photogenerated exciplexes, which partially dissociated to form CS states. In contrast, a slow recombination of the separated charge carriers leads to LPL for more than 2 h with a power-law decay profile, and its duration is dependent on the excitation time, sample temperature, and the content of embedded m-CDs. Moreover, the small values of ΔE(^1^LE_D_-^1^CT) and ΔE(^1^CT-^3^LE_D_) might trigger excitons to transfer from ^1^CT to ^1^LE_D_ (RISC) and to ^3^LE_D_ (ISC), respectively, and then radiatively relax to the ground state. Consequently, the LPL emission of m-CDs@CA may consist of TADF from ^1^LE_D_, PL from ^1^CT, and phosphorescence from ^3^LE_D_ initiated from CS states of the exciplex (Fig. 5b), which are in good accordance with the LPL spectra shown in Fig. 4a (bottom).

### Applications of long persistent luminescence emissive m-CDs@CA

**Fig. 6:**
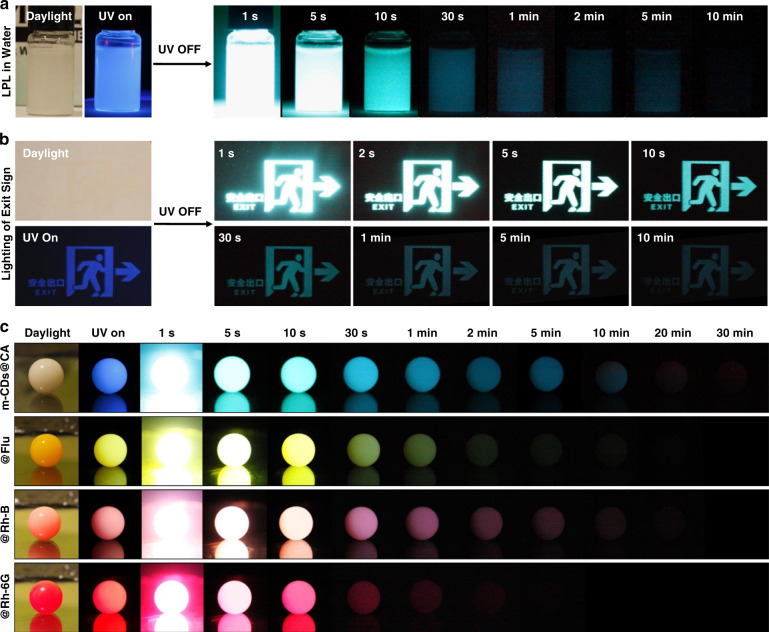
The robust LPL property of m-CDs@CA and its possible applications. **a** Photographs of the aqueous m-CDs@CA dispersion in daylight, under UV lamp (365 nm) and after the UV lamp was turned off after 1 min irradiation. **b** Images of an emergency sign after UV irradiation was stopped; the sign was created using an m-CDs@CA suspension patterned on filter paper by silk-screen printing. **c** Photographs of luminous pearls prepared using m-CDs@CA with and without added fluorescent dyes (dispersed in epoxy resin) under daylight, a UV lamp (365 nm) and switching off the UV lamp after 1 min of irradiation.

In general, the LPL emission of organic exciplex systems is highly related to the photoinduced CS states, which are unstable in the presence of water or oxygen^19–21^. As a result, encapsulation in an inert atmosphere and/or harsh rigidification of the exciplexes are required to achieve LPL. Importantly, m-CDs@CA exhibits rarely observed LPL under ambient conditions, even in aqueous media (Fig. 6a). As shown in Fig. S15 in the supplementary information, the afterglow intensities were observed to be nearly the same for m-CDs@CA powder under air and argon atmospheres, demonstrating that the CA crystal plays a critical role in protecting the excited states of m-CDs@CA from being quenched by oxygen. The remaining LPL in aqueous media might be due to the ability of CA to form a hydrogen-bond network and the strong covalent bonds between m-CDs and CA, which effectively stabilize the CS and CT states and prevent quenching by oxygen and water^40,41,48^. Moreover, m-CDs@CA also exhibits excellent photostability and storage stability (Figs. S16-S17 in the supplementary information). Due to such superior LPL features, m-CDs@CA are promising in many fields of application. For instance, this material could be conveniently patterned on a flexible substrate such as filter paper for lighting emergency exits without a continuous power supply (Fig. 6b). Moreover, luminous pearls can be facilely fabricated by dispersing m-CDs@CA in epoxy resin. Upon 365 nm UV light excitation, the luminous pearl displayed bright blue LPL for more than 30 min (Fig. 6c). Furthermore, multicolor luminous pearls can also be fabricated using the same method by adding an appropriate fluorescent dye such as fluorescein (Flu), rhodamine 6 G (Rh-6G), or rhodamine B (Rh-B) (Fig. 6c). Such multicolored LPL can be attributed to the Förster resonance energy transfer (FRET) between the m-CDs@CA and the fluorescent dyes. The FRET efficiency is strongly dependent on the molar extinction coefficients of dyes (Table S7 in the supplementary information) and the overlap of the LPL emission of m-CDs@CA and the absorptions of different dyes (Fig. S18 in the supplementary information). Different durations of multicolor luminous pearls were observed.

## Discussion

In summary, a robust OLPL system with hour-level emission was developed in this study via facile microwave-assisted heating of a mixture of m-CDs and urea. To the best of our knowledge, this is a very rare example of an OLPL system that is applicable under ambient conditions and even in aqueous media. Further studies demonstrated that CA was produced from urea in situ during the microwave heating process and bonded with m-CDs by forming C–N covalent bonds. The origin of LPL of m-CDs@CA has been attributed to the recombination of CS states of exciplexes produced from m-CDs and CA upon UV light irradiation. In addition, the formation of a C–N covalent bond between m-CDs and CA is confirmed to play a critical role in activating LPL of m-CDs@CA, which, with the assistance of hydrogen bonds and the physical confinement of the CA matrix, effectively stabilizes the CS states of the exciplexes and prevents quenching by water and oxygen. Finally, applications of glow-in-the-dark m-CDs@CA, such as emergency signs and multicolored luminous pearls, were demonstrated. This study developed a facile strategy of preparing OLPL materials that are applicable under ambient conditions, which could not only effectively expand the scope of CD-related research and applications but also offer a new idea for designing OLPL systems with robust features.

## Materials and method

### Materials

The syntheses of m-CDs, m-CDs@CA, and experimental cyanuric acid (i.e., eCA) were referenced in our previous reports^28,49^. The co-crystallized composite (i.e., m-CDs#CA) was prepared according to a literature method^47^. The processes for the preparation of these materials are also supplied in the experimental section in the supplementary information.

### Preparation of the luminous pearls

Typically, 200 mg of m-CDs@CA powder was first mixed with 3.0 g of epoxy resin B and then diluted with 9.0 g of epoxy resin A. To obtain different colored luminous pearls, different fluorescent dyes (100 mg) (e.g., fluorescein (Flu), rhodamine 6 G (Rho 6 G), or rhodamine B (Rho B)) were employed as luminescent dopants. Finally, luminous pearls with azure-, olivine-, pink-, and red-colored LPL emission were prepared by filling well-mixed suspensions into a ball-shaped mold and standing for 36 h.

### Preparation of the emergency ink

First, 200 mg of the fine powder of m-CDs@CA was mixed with 10 mL of commercial ink and then well mixed by mechanical stirring for 30 min. The obtained suspension can be employed as an LPL ink for silk-screen printing.

### Determination of the energy level and band gap

The LUMO and HOMO energy levels of m-CDs and CA were calculated on the basis of cyclic voltammetry (CV) measurements, which were carried out on a bioanalytical system (BAS 100 W). In these experiments, a glass-carbon disk electrode, Pt wire, and Ag/Ag^+^ were used as the working electrode, counter electrode, and reference electrode, respectively. The energy levels of the HOMO and LUMO are calculated according to the following empirical formulas:

$$\begin{array}{*{20}{c}} {E_{{\mathrm{HOMO}}}\left( {{\mathrm{eV}}} \right) = - \left( {\varphi _{{\mathrm{ox}}} + 4.71} \right)} \\ {E_{{\mathrm{LUMO}}}\left( {{\mathrm{eV}}} \right) = - \left( {\varphi _{{\mathrm{red}}} + 4.71} \right)} \end{array}$$

where *E*_*HOMO*_ and *E*_*LUMO*_ are the energy levels of the HOMO and LUMO, respectively, and *φ*_*ox*_ and *φ*_*red*_ are the oxidation onset potential and initial reduction potential of the materials, respectively.

The energy levels of different excited states (energy gap between them and the ground state) were calculated based on the following empirical formula:

$${\Delta}E\left( {{{{\mathrm{eV}}}}} \right) = 1240/\lambda _{EM}\left( {{{{\mathrm{nm}}}}} \right)$$

where Δ*E* is the energy level of different excited states (the singlet (^1^LE_D_), triplet (^3^LE_D_), and charge transfer (^1^CT) states), λ_EM_ is the wavelength of the FL and phosphorescence emission peaks at low temperature (77 K), and LPL emission peak (470 nm) of m-CDs@CA. The calculated energy levels and bandgaps of m-CDs (a) and pCA are summarized in Table S6 (supplementary information).

## Supplementary information

supplementary information
