# Supplementary material for: Enabling robust and hour-level organic long persistent luminescence from carbon dots by covalent fixation
Source: Light Sci Appl. 2022 Mar 29;11:80. doi: 10.1038/s41377-022-00767-y (PMC8964705; doi:10.1038/s41377-022-00767-y)
Supplement: Supplementary file 1 — supplementary information [file 41377_2022_767_MOESM1_ESM.doc]

**Supplementary Information for**

Enabling Robust and Hour-level Organic Long Persistent Luminescence from Carbon Dots by Covalent Fixation

Kai Jiang,^1,*^ Yuci Wang,^2^ Cunjian Lin,^3^ Licheng Zheng,^1^ Jiaren Du,^1^ Yixi Zhuang,^3^ Rongjun Xie,^3^ Zhongjun Li,^2^ and Hengwei Lin^1,*^

^1^International Joint Research Center for Photo-responsive Molecules and Materials, School of Chemical and Material Engineering, Jiangnan University, Wuxi 214122, China

^2^College of Chemistry, Zhengzhou University, Zhengzhou 450001, China

^3^State Key Laboratory of Physical Chemistry of Solid Surface, Fujian Provincial Key Laboratory of Materials Genome, and College of Materials, Xiamen University, Xiamen 361005, China

^*^Correspondence: Kai Jiang (Tel: 0086-574-8668-8163, E-mail: jiangkai@nimte.ac.cn) and Hengwei Lin (Tel: 0086-510-85917090, E-mail: linhengwei@jiangnan.edu.cn).

**Experimental Section**

**Reagents**

Reagent grade of m-phenylenediamine (mPD), fluorescein, rhodamine B and rhodamine 6G were bought from Aladdin Chemicals Co. Ltd (Shanghai, China). Methylene chloride, methanol and ethanol were purchased from Sinopharm Chemical Reagent Co. Ltd (Shanghai, China). Pure cyanuric acid (pCA) was purchased from J&K Chemical Reagent Co. Ltd (Beijing, China). All chemicals were used as received without further purification unless otherwise specified. Deionized (DI) water was used throughout this study.

**Preparation of m-CDs@CA**

To prepare the composite materials with different loading amounts of m-CDs, 5 mg, 10 mg, 50 mg, 100 mg, 200 mg or 1000 mg of m-CDs were added into 20 mL of urea solution (0.5 g/mL, total 10 g) to form a transparent solution. Subsequently, the mixture solution was transferred into a beaker and heated in a domestic oven for 8-10 min (750 W) until the water completely evaporated. The formed crude composites were purified by grinding into powder, dispersed by boiled water (100 mL) and centrifuged at 5000 rpm for 5 min to remove the insoluble impurities. Finally, the m-CDs@CA were gradually separated out with the solution cooling to room temperature and then dried in a vacuum oven (60 ℃ for 12 h).

**Synthesis of experimental cyanuric acid (eCA)**

Typically, 10 g urea was dissolved in 20 mL of DI water, and the formed transparent solution was transferred into a beaker and heated in a domestic oven for 8-10 min (750 W). The crude product was crushed and purified by dispersing in boiling water (100 mL) and centrifuging at 5000 rpm for 5 min to remove the insoluble components. The pure product was gradually separated out with the solution cooling down to room temperature, and further dried in a vacuum oven (60 °C for 12 h).

**Preparation of m-CDs#CA**

In brief, 400 uL of m-CDs (5 mg/mL in ethanol) were added into a clear and transparent solution (5 mL of boiled water) of pCA (0.5 g). The mixture solution allowed to cool down to room temperature and the crystals were gradually separated out within 6 h. The obtained m-CDs#CA crystals were washed with DI water (20 mL) and then subjected to freeze drying.

**Characterization**

Transmission electron microscopy (TEM) observations were performed on a Tecnai F20 microscope. Scanning electron microscopy (SEM) was performed on a JEOL FESEM 6700F microscope with a primary electron energy of 3 KV. X-Ray powder diffraction (XRD) patterns were recorded on a Rigaku D/max-2000 X-ray powder diffractometer (XRD) (Japan) using Cu Kα (1.5406 Å) radiation. X-ray photoelectron spectroscopy (XPS) spectra were carried out with ESCALAB 250Xi (Thermo Scientific). Fourier transform infrared (FT-IR) spectra were obtained on a Nicolet 6700 FT-IR spectrometer. Time-resolved electron spin resonance (ESR) signals were tested on a Bruker E500 spectrometer, and the samples were excited using 365 nm UV lamp. Photoluminescence (PL), afterglow emission and excitation spectra were measured on a Hitachi F-4600 spectrophotometer at ambient conditions. UV-Vis absorption spectra were recorded on a PERSEE T10CS UV-Vis spectrophotometer. PL and afterglow lifetimes were measured using Fluorolog 3-11 (HORIBA Jobin Yvon). PL quantum yields (QYs) were measured on a QE-2100 quantum efficiency measurement system (Japan Otsuka Electronics). Photographs of PL and afterglow were taken using a Canon camera (EOS 550) under excitation by a hand-hold UV lamp.

**Supplementary data**

**Figure S1.** FL emission spectra at different excitation wavelengths and excitation spectra at emission of 430 nm and/or 470 nm of m-CDs@CA powder with different ratio of m-CDs at ambient conditions.

**Figure S2.** Afterglow emission spectra at different excitation wavelengths and excitation spectra at emission of 430 nm and/or 470 nm of m-CDs@CA powder with different ratio of m-CDs at ambient conditions.

**Figure S3.** SEM image of the m-CDs@CA.

**Figure S4.** a) SEM and b) high-resolution of SEM images of pCA.

**Figure S5.** TEM (a) and high resolution TEM (b) images of m-CDs.

**Figure S6.** PL emission spectra at different excitation wavelengths and excitation spectrum at emission of 430 nm of m-CDs (ethanol dispersion).

**Figure S7.** Afterglow (TADF) decay spectrum and fitting curve of the m-CDs@CA powder under the excitation of 350 nm (λ_em_=430 nm).

**Figure S8.** Electron spin resonance (ESR) spectra of the m-CDs@CA before and after UV light (365 nm) irradiation.

**Figure S9.** The PL emission spectra under different excitation wavelengths and the excitation spectrum under the emission of 390 nm of m-CDs#CA powder.

**Figure S10.** Afterglow emission spectra under different wavelengths and excitation spectra under the emission of 425 nm and 480 nm of m-CDs#CA powder.

**Figure S11.** TADF and RTP decay spectra and fitting curve of m-CDs#CA powder under the excitation of 320 nm (λ_em_(TADF)=425 nm, λ_em_(RTP)= 480 nm).

**Figure S12.** Low temperature (77 K) fluorescence (black line) and phosphorescence (red line) emission spectra of m-CDs dispersed in ethanol.

**Figure S13.** Low temperature (77 K) fluorescence (black line) and phosphorescence (red line) emission spectra of m-CDs@CA powder.

**Figure S14.** Cyclic voltammograms of m-CDs (a) and pCA (b) in DMF solution.

**Figure S15.** Afterglow emission spectra of m-CDs@CA powder under air (Air) and argon (Ar) atmospheres at 365 nm excitation.

**Figure S16.** Photostability of the m-CDs@CA powder under continuous excitation at 365 nm (emission wavelength of 480 nm) for one hour using spectrofluorometer equipped with a xenon lamp (150 W).

**Figure S17.** Comparison of the afterglow emission of the freshly-prepared and the stored (4 months) m-CDs@CA (powder) under excitation of 365 nm.

**Figure S18.** The overlap of the LPL emission of m-CDs@CA (λ_EX_=365 nm) and the absorptions of different dyes.

**Table S1**. The PLQYs of m-CDs@CA powder with different ratio of m-CDs.

| **Ratio** | 0.05 wt% | 0.1 wt% | 0.5 wt% | 1 wt% | 2 wt% | 10 wt% |
| --- | --- | --- | --- | --- | --- | --- |
| **QYs** | 39.03% | 35.48% | 14.43% | 15.23% | 13.24% | 0.556% |

**Table S2.** Fitted parameters of the TADF decay curve of m-CDs@CA powder under excitation of 350 nm.

| **λ_ex_(nm)** | **λ_em_**  **(nm)** | **τ_1_**  **(ms)** | **B_1_(%)** | **τ_2_**  **(ms)** | **B_2_(%)** | **τ_3_**  **(ms)** | **B_3_(%)** | **τ_avg_(s)** | ***ϕ*** |
| --- | --- | --- | --- | --- | --- | --- | --- | --- | --- |
| 350 | 430 | 89.03 | 5.84 | 756.54 | 49.22 | 1492.17 | 44.94 | **1.223** | 1.093 |

**Table S3.** Fitted parameters of the TADF and RTP decay curves of m-CDs#CA powder under excitation of 350 nm.

| **λ_em_(nm)** | **τ_1_(ms)** | **B_1_(%)** | **τ_2_(ms)** | **B_2_(%)** | **τ_3_(ms)** | **B_3_(%)** | **τ_avg_(s)** | ***ϕ*** |
| --- | --- | --- | --- | --- | --- | --- | --- | --- |
| 430 | 2.25 | 0.27 | 245.34 | 4.86 | 1666.63 | 94.88 | **1.656** | 1.1641 |
| 480 | 16.98 | 2.75 | 206.10 | 12.12 | 1563.07 | 85.13 | **1.538** | 1.1815 |

**Table S4.** Relative contents of C, N and O elements of the m-CDs, pCA, m-CDs@CA and m-CDs#CA on the basis of the XPS data.

| **Sample** | **C (％)** | **N (％)** | **O (％)** |
| --- | --- | --- | --- |
| **m-CDs** | 79.17 | 1.46 | 19.37 |
| **pCA** | 38.16 | 38.23 | 23.61 |
| **eCA** | 37.58 | 38.17 | 24.25 |
| **m-CDs@CA** | 40.21 | 31.64 | 28.15 |

**Table S5.** Relative contents of different functional groups in the m-CDs, pCA and m-CDs@CA based on the HR XPS fittings.

| **Sample** | **C 1s** | | | |  | **N 1s** | | | | |  | **O 1s** | |
| --- | --- | --- | --- | --- | --- | --- | --- | --- | --- | --- | --- | --- | --- |
|  | **C-C/C=C** | **C-N** | **C-O** | **N-C=N** |  | **Pyridinic**  **N** | **Amino**  **N** | **N-(C)_3_** | **Pyrrolic N/**  **C=N-C** | **N-H** |  | **C=O** | **-OH** |
| **m-CDs** | 85.36 | 11.13 | 3.51 | - |  | 39.29 | 39.95 | - | 20.76 | - |  | - | 100 |
| **pCA** | 12.09 | 6.59 | - | 81.32 |  | - | - | - | 88.85 | 11.15 |  | 56.67 | 43.33 |
| **m-CDs@CA** | 16.91 | 9.29 | - | 73.80 |  | 7.37 | 21.88 | 14.82 | 49.74 | 6.19 |  | 74.39 | 25.61 |

**Table S6.** Photophysical properties of the donor (m-CDs), acceptor (pCA), and exciplex system (m-CDs@CA).

| **Sample** | **HOMO [eV]** | **LUMO [eV]** | ***Φ*_PL_** | **τ_FL_ [ns]** | **τ_Phos_/τ_TADF_ [s]** | **^1^LE_D_/^1^LE_A_ /^1^CT [eV]** | **^3^LE_D_/ ^3^LE_A_/^3^CT [eV]** |
| --- | --- | --- | --- | --- | --- | --- | --- |
| **m-CDs** | -5.775 |  | 4.8 | 6.41 | 0.456 | 2.96 | 2.54 |
| **pCA** | -6.091 | -3.361 |  |  |  |  |  |
| **m-CDs@CA** |  |  | 35.48 | 4.9 | 1.223 | 2.64 |  |

**Table S7.** The calculated molar extinction coefficients of fluorescein (Flu), rhodamine 6G (Rh-6G) and rhodamine B (Rh-B).

|  | **C (mol L^-1^)** | **L (cm)** | **A (a. u.)** | **λ (nm)** | **ε (L mol^-1^ cm^-1^)** |
| --- | --- | --- | --- | --- | --- |
| **Flu.** | 0.15045 | 1 | 0.12 | 486 | 797.6 |
| **Rho B** | 0.10435 | 1 | 1.74 | 546 | 1667.5 |
| **Rho 6G** | 0.10435 | 1 | 0.97 | 530 | 929.4 |
